# Mesenchymal cell survival in airway and interstitial pulmonary fibrosis

**DOI:** 10.1186/1755-1536-3-15

**Published:** 2010-08-25

**Authors:** James C Bonner

**Affiliations:** 1 Department of Environmental and Molecular Toxicology, North Carolina State University, Raleigh, North Carolina 27695, USA

## Abstract

Fibrotic reactions in the airways of the lung or the pulmonary interstitium are a common pathologic outcome after exposure to a wide variety of toxic agents, including metals, particles or fibers. The survival of mesenchymal cells (fibroblasts and myofibroblasts) is a key factor in determining whether a fibroproliferative response that occurs after toxic injury to the lung will ultimately resolve or progress to a pathologic state. Several polypeptide growth factors, including members of the platelet-derived growth factor (PDGF) family and the epidermal growth factor (EGF) family, are prosurvival factors that stimulate a replicative and migratory mesenchymal cell phenotype during the early stages of lung fibrogenesis. This replicative phenotype can progress to a matrix synthetic phenotype in the presence of transforming growth factor-β1 (TGF-β1). The resolution of a fibrotic response requires growth arrest and apoptosis of mesenchymal cells, whereas progressive chronic fibrosis has been associated with mesenchymal cell resistance to apoptosis. Mesenchymal cell survival or apoptosis is further influenced by cytokines secreted during Th1 inflammation (e.g., IFN-γ) or Th2 inflammation (e.g., IL-13) that modulate the expression of growth factor activity through the STAT family of transcription factors. Understanding the mechanisms that regulate the survival or death of mesenchymal cells is central to ultimately developing therapeutic strategies for lung fibrosis.

## Background

**Figure 1:**
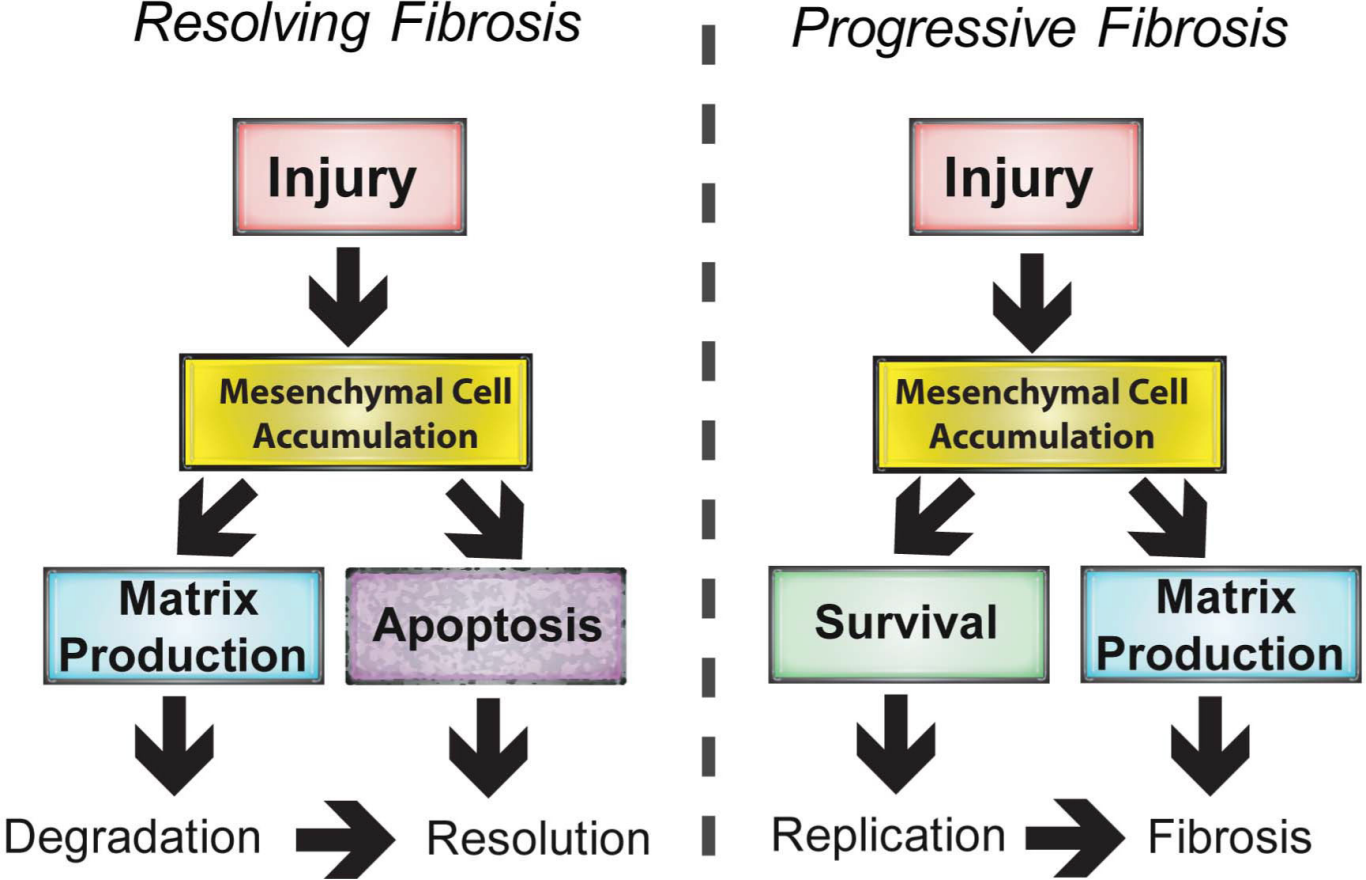
**Comparison of events that mediate "resolving fibrosis" versus "progressive chronic fibrosis**." Following injury, mesenchymal cell accumulation and subsequent matrix (e.g., collagen) production occur in both resolving and progressive fibrogenesis. However, mesenchymal cell apoptosis and degradation of matrix are favored in resolving fibrosis, whereas mesenchymal survival is favored in progressive fibrosis. Mesenchymal cell survival is likely caused by enhanced growth factor responsiveness and resistance to apoptosis.

Fibrosis is a feature of many environmental and occupational lung diseases where pathological changes occur either around the conducting airways [1] or within the pulmonary interstitium of the distal lung parenchyma [2]. In many instances, the insulting agent causes a sustained and progressive fibroproliferative response that compromises lung function. In chronic fibrosis, including asbestosis [2,3], sarcoidosis [4] and idiopathic pulmonary fibrosis (IPF), mesenchymal cell survival and resistance to apoptosis favor the development of progressive disease that ultimately results in respiratory failure [5,6]. However, in other instances, the mesenchymal cell proliferative response to tissue injury by inhaled agents resolves to varying degrees. For example, asthma features airway fibrosis, but the lesions are relatively confined to the distal airways and fibrogenesis generally does not progress to the lung parenchyma [7,8]. Similarly, the transition metal vanadium pentoxide released from oil-burning power plants is a cause of occupational chronic bronchitis resulting in reduced airway function in workers, yet this disease rarely progresses to chronic interstitial fibrosis [9]. The precise cellular and molecular mechanisms that initiate fibrogenesis in the lung can be quite varied and depend on the insulting agent (e.g., metals, fibers, chemotherapeutic drugs, radiation). Genetic susceptibility also plays a major role in determining disease progression. Despite the complexities of gene-environment interactions that serve to initiate lung fibrogenic reactions, a common denominator that is central to the progression of fibrosis is airway and interstitial mesenchymal cells that provide the major source of secreted collagen that defines end stage lung fibrosis. The term *mesenchymal cell *is used throughout this review and includes several phenotypes (fibroblasts, myofibroblasts, smooth muscle cells and fibrocytes). There is also considerable plasticity among the mesenchymal cell phenotypes. For example, fibroblasts are known to differentiate into myofibroblasts in the presence of transforming growth factor (TGF)-β1. The most notable mesenchymal phenotype that contributes the majority of secreted matrix during the fibrogenic process is the myofibroblast [9]. Abundant evidence indicates that myofibroblasts provide the major source of collagen that defines the fibrotic lesion and that TGF-β1 is the dominant growth factor that stimulates matrix synthesis by lung mesenchymal cells [2,9]. Because myofibroblasts are the central source of extracellular matrix, the survival of these cells largely determines overall disease progression. Mesenchymal cell survival in the lung is a key determinant of whether fibrosis will progress or resolve. Whether the proliferative response to injury ultimately resolves through mesenchymal cell growth arrest and apoptosis or whether mesenchymal cell survival is sustained to perpetuate chronic and persistent matrix production is the central topic of this review. The overall premise of resolving versus progressive fibrosis is illustrated in Figure 1. In both resolving and progressive fibrogenic scenarios, mesenchymal cell accumulation can result from several possible mechanisms (recruitment of circulating fibrocytes, epithelial-mesenchymal cell transition, or migration and proliferation of resident lung mesenchymal cells). However, in resolving fibrosis, the collagen matrix deposited by mesenchymal cells is degraded by protease activity such as matrix metalloproteinases and is also ultimately limited by mesenchymal cell growth arrest and apoptosis. In contrast, progressive fibrosis is the result of sustained matrix deposition or lack of matrix degradation, coupled with mesenchymal cell survival. Mesenchymal cell survival is likely due to multiple factors, including enhanced or sustained responsiveness of these cells to growth factor signals and the resistance of mesenchymal cells to apoptosis.

### Mesenchymal Cell Survival: Enhanced Growth Factor Responsiveness and Resistance to Apoptosis

The survival of mesenchymal cells is likely due in part to enhanced responsiveness to growth factors and cytokines that stimulate migration and proliferation or reduce apoptosis. Enhanced responsiveness to proliferative and matrix synthetic signals has been reported in fibroblasts from patients with idiopathic pulmonary fibrosis (IPF). For example, pulmonary fibroblasts from IPF patients have spontaneously elevated levels of IL-13 and IL-4 receptor subunits, and it has been suggested that the abnormal proliferative properties of lung fibroblasts from certain lung fibrosis patient groups can be modulated in a manner that is dependent on the IL-4 and IL-13 receptor expression [10]. Additionally, IPF fibroblasts stimulated with exogenous TGF-β1, interleukin (IL)-13 or CC-chemokine ligand 2 (CCL2) have significantly increased levels of connective tissue growth factor (CTGF), TGF-β1, and cell-surface receptors for TGF-β1, IL-13 and platelet-derived growth factor (PDGF) [11]. This suggests that enhanced responsiveness of lung fibroblasts from IPF patients is likely due to a complex interplay between cytokines, growth factors and elevated levels of several different cell-surface receptors.

A major factor that determines mesenchymal cell survival and the severity of a fibrogenic response is the resistance of mesenchymal cells to undergo apoptosis after injury. Myofibroblasts undergo apoptosis during normal wound healing as a way to limit scar formation in multiple tissues, including lung, liver and kidney [12,13]. During excessive scarring, i.e., fibrosis, it has been suggested that the process of mesenchymal cell apoptosis cannot take place or is severely reduced [13]. Resistance to apoptosis has been reported in cultured lung myofibroblasts isolated from patients with IPF, and resistance to apoptosis could be due to altered IL-6 signaling [14]. Specifically, IL-6 protects against Fas-induced apoptosis in IPF fibroblasts, and yet it enhances the apoptotic effect of Fas in normal fibroblasts. These contrasting effects of IL-6 in normal versus IPF lung fibroblasts appear to be due to altered cell signaling involving MAP kinase and STAT-3 transcription factor. Other factors also likely contribute to the resistance of mesenchymal cells to apoptosis during fibrogenesis. For example, patients with IPF have a diminished capacity to produce prostaglandin (PG)E_2_, which results in increased sensitivity of alveolar epithelial cells to Fas ligand-induced apoptosis but induces fibroblast resistance to the same stimulus [15].

### Epithelial-Mesenchymal Cell Interactions in Lung Fibrogenesis

In contrast to the resistance of mesenchymal cells in IPF, epithelial cell apoptosis is widespread [16]. Therefore, the apoptosis paradox in fibrosis is that epithelial cells are sensitive to apoptosis during the disease process, while mesenchymal cells are resistant to apoptosis. The airway epithelium serves multiple functions, including protection against inhaled toxicants, clearance of particles and fibers from the lung via the mucociliary apparatus, and repair processes mediated by soluble cytokines, growth factors, lipid mediators and proteinases [17]. Dramatic changes to the architecture of the airway walls occur as a result of epithelial injury in patients with asthma, cystic fibrosis and chronic obstructive pulmonary disease (COPD) [18]. Likewise, injury to type I epithelial cells of the alveolar region plays a critical role toward initiating interstitial lung fibrosis [5]. Because of the many protective and homeostatic functions of the airway epithelium, damage to the epithelial lining and subsequent apoptosis plays a major role in fibrogenesis if adequate repair does not occur following injury. As such, there is a constant struggle within the airway microenvironment to repair sites of injured epithelium while limiting mesenchymal cell activity and matrix deposition. In general terms, the progression of lung fibrosis is favored by the combination of epithelial cell death and mesenchymal cell survival. The recovery of an intact epithelium following lung injury is critical for restoration of lung homeostasis [19]. Failure to repair the epithelial barrier promotes mesenchymal cell survival and matrix production. Some growth factors, including members of the epidermal growth factor (EGF) family, discussed in more detail below, can play dual roles in repairing injured epithelium and yet also stimulate mesenchymal cell survival.

Proper communication between epithelial cells lining the airways and the underlying mesenchymal cells is critical for maintaining normal tissue function and homeostasis in the lung. The structure that comprises the airway epithelium and the underlying mesenchymal tissue and extracellular matrix has been referred to as the epithelial-mesenchymal cell trophic unit (EMTU), and structure-function relationships between EMTU elements has been most extensively applied to evolving theories on the pathogenesis of asthma [20]. However, these EMTU structure-function relationships also apply to other chronic airway diseases such as COPD as well as interstitial lung diseases of the alveolar region that include asbestosis, silicosis and IPF.

**Figure 2:**
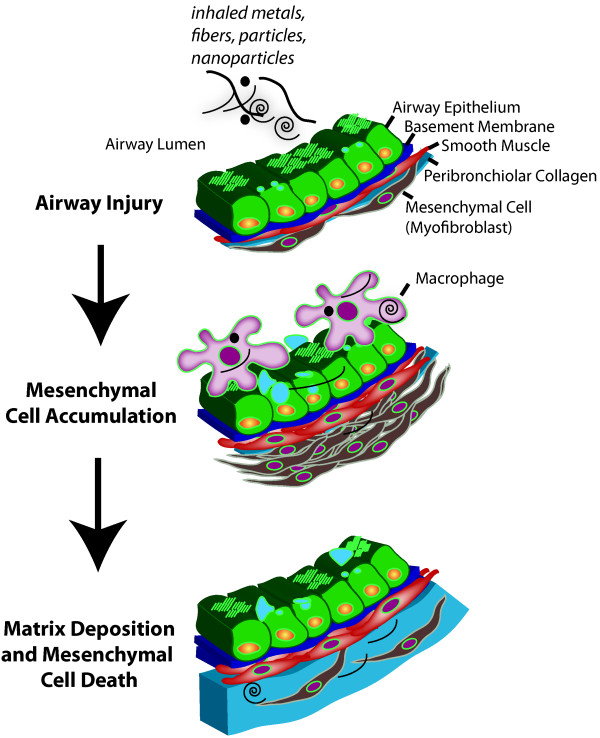
**Mesenchymal cell survival in the progression of airway fibrosis following injury by environmental factors**. Airway injury occurs after the inhalation of a variety of inhaled metals, fibers, particles or nanoparticles. After initial injury by particle insult, the airway epithelium produces chemokines that attract macrophages. Both macrophages and the epithelium produce cytokines, chemokines and growth factors that stimulate mesenchymal cell (fibroblast and myofibroblast) accumulation within days. Airway smooth muscle also undergoes hyperplasia and hypertrophy after particle injury. Mesenchymal cells undergo growth arrest and apoptosis, but leave deposited extracellular matrix (e.g., collagen) that defines the airway fibrotic lesion.

Rodent models of fibrotic airway and interstitial lung diseases have been extremely valuable in elucidating mechanisms of epithelial-mesenchymal cell interaction and formulating new ideas related to the importance of the EMTU in lung fibrosis. For example, vanadium pentoxide (V_2_O_5_)-induced airway injury is a useful rodent model to study the relationship between airway epithelial cell activation and differentiation in the context of mesenchymal cell survival and fibrosis [21]. Lung injury caused by a single administration of V_2_O_5 _is followed by a multistep fibrogenic process that consists of (1) epithelial cell activation and differentiation, macrophage accumulation and mesenchymal proliferation; and (2) collagen production by the mesenchymal cells followed by apoptosis, which serves to resolve the fibrogenic response. Similar pathologic events are seen in a murine model of allergic airway disease caused by sequential exposure to ovalbumin and nanoparticles [22]. The common pathological features of airway remodeling caused by a partially resolving fibrogenic response to oxidative stress from metals, fibers, particles or nanoparticles are illustrated in Figure 2. In both of these scenarios, the airway epithelium is activated to differentiate from a ciliated, serous cell phenotype to a hypersecretory epithelium. Epithelial differentiation is accompanied by mesenchymal cell accumulation and proliferation around airways. Mesenchymal cells become activated to secrete a collagen matrix. However, the fibrogenic process is partially resolved in that the majority of myofibroblasts disappear, presumably through apoptotic pathways.

Tissue homeostasis within the EMTU is tightly regulated by a multiplicity of secreted factors produced by the epithelium, infiltrating inflammatory cells and the underlying mesenchymal cells. It is also likely that physical contact between epithelial cells and mesenchymal cells is important to maintaining normal airway architecture as dendritic processes of subepithelial mesenchymal cells have been demonstrated to contact the epithelial basement membrane [23]. Physical contact between epithelium and mesenchymal cells is likely disrupted during fibrogenesis by deposited extracellular matrix. The epithelium secretes growth factors (e.g., HB-EGF, TGF-α) that serve to repair the epithelial barrier after injury, and yet these same factors promote survival, replication, and migration of subepithelial mesenchymal cells [8]. These secreted growth factors are important to tissue homeostasis and repair but also play important roles in fibrogenesis when their expression or signaling is dysregulated.

### The PDGF Family: Prosurvival Factors for Mesenchymal Cells

**Figure 3:**
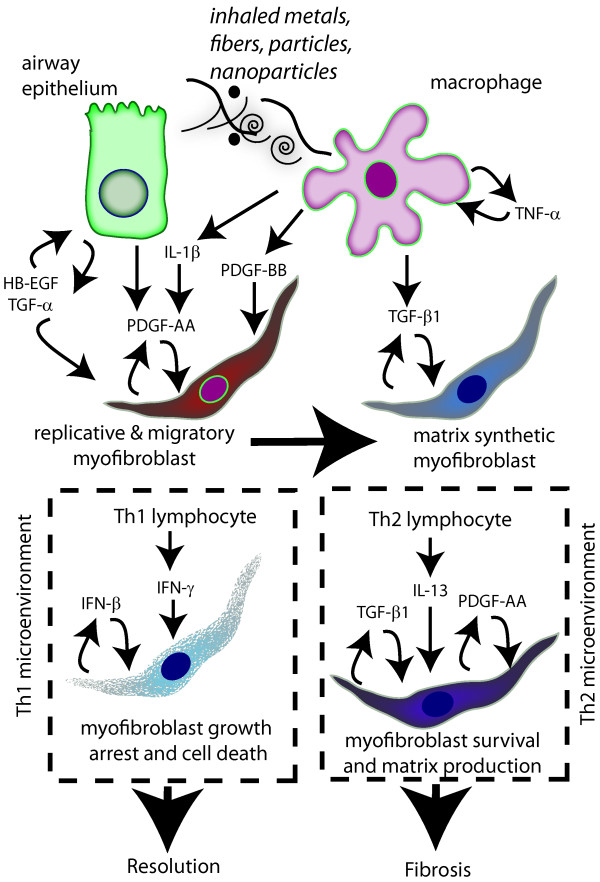
**Growth factors and cytokines from macrophages and epithelial cells, along with the inflammatory microenvironment, influence mesenchymal cell survival and phenotype**. Particle-stimulated airway epithelial cells and alveolar macrophages produce soluble cytokines and growth factors that stimulate the replication and migration of myofibroblasts. EGFR ligands (HB-EGF and TGF-α) are produced by airway epithelial cells that stimulate epithelial repair and differentiation in an autocrine manner, but also stimulate myofibroblast replication. PDGF-AA and PDGF-BB produced by epithelial cells and macrophages, respectively, drive replication and chemotactic migration of myofibroblasts. IL-1β enhances the activity of PDGF-AA and PDGF-BB by upregulating PDGF receptor-α (PDGFRα) expression. Macrophages are also an abundant source of TNF-α, which stimulates TGF-β1 production by macrophages in an autocrine manner. TGF-β1 stimulates myofibroblast collagen production and growth arrest, which defines a "matrix synthetic phenotype." The myofibroblast phenotype is further defined by a Th1 or Th2 microenvironment. In the presence of Th1 lymphocytes, IFN-γ produced by Th1 cells or IFN-β produced by myofibroblasts stimulates growth arrest and cell death of myofibroblasts and leads to the resolution of a fibrogenic response and tissue repair. In the presence of Th2 lymphocytes, IL-13 produced by Th2 cells activates myofibroblasts to produce PDGF-AA and TGF-β1 in an autocrine manner, which drives myofibroblast survival, replication and matrix production to enhance and sustain a fibrogenic response.

The mesenchymal cell response to injury by fibrogenic agents is mediated by a variety of secreted factors (cytokines, chemokines, and growth factors) that activate intracellular signaling pathways through their cognate receptors. The cell types that serve as potential sources of these soluble mediators to influence mesenchymal cell fate are diverse and include epithelial cells, mononuclear phagocytes (e.g., macrophages), lymphocytes, and mesenchymal cells themselves. As illustrated in Figure 3, a variety of toxic metals and metal-containing particles and fibers activate airway epithelial cells and macrophages to secrete cytokines (e.g., IL-1β, TNF-α) and growth factors (e.g., HB-EGF, PDGF) that stimulate myofibroblast replication and chemotaxis (directed migration). These cells also produce TGF-β1 that stimulates or activates the transition of fibroblasts from a "replicative and migratory" phenotype to a "matrix synthetic" myofibroblast phenotype [24].

Platelet-derived growth factor (PDGF) is a key factor in the survival and differentiation of mesenchymal cells during lung development, and PDGFs are also important for tissue repair following injury in adult tissues. However, overexpression of PDGF or its receptors is thought to play a pivotal role in the progression of fibrotic diseases [25]. The cellular responses to PDGF signaling include proliferation, migration, control of differentiation, and survival [25,26]. There are four PDGF genes, designated A-D, that encode four homodimeric protein isoforms (PDGF-AA, -BB, -CC, and -DD) and one heterodimeric isoform (PDGF-AB). There are also two PDGF receptors, PDGF-Rα and PDGF-Rβ, that dimerize upon ligand binding, forming three isoforms (PDGF-Rαα, -Rαβ, and -Rββ). PDGF-AA and PDGF-CC bind exclusively to PDGF-Rα, whereas PDGF-BB, -AB, and -DD isoforms bind both PDGF-Rα and PDGF-Rβ [27]. PDGF activates multiple intracellular signaling molecules that play important roles in mesenchymal cell survival, including MAP kinases and the STAT family members STAT-1 and STAT-3.

Abundant evidence indicates that PDGF and its receptors are important in mediating the pathogenesis of airway and interstitial lung fibrosis [25]. First, PDGF ligands are elevated in patients with idiopathic pulmonary fibrosis, and immunohistochemical studies have shown that increased expression of PDGFs occurs at sites of fibroproliferative lesions [28]. Second, the expression of PDGF and its receptors are increased in lung tissue during the mesenchymal cell proliferative phase of pulmonary fibrosis in rodent models where injury is induced by agents such as bleomycin [29,30], asbestos [31,32], metals [33] or nanoparticles [34,35]. Third, PDGFs are potent mitogens and chemoattractants for mesenchymal cells in lung and other organ systems [25], and PDGF receptor activation is essential for mesenchymal cell migration in wound healing [36]. Fourth, PDGF is produced by lung macrophages, epithelial cells and mesenchymal cells *in vitro *following stimulation with particles or fibers [37,38]. As illustrated in Figure 3, PDGF ligands secreted by epithelial cells and macrophages contribute to the replicative and migratory myofibroblast phenotype. Finally, transgenic mouse studies demonstrate critical roles for PDGF in mesenchymal cell survival in the lung. Knockout mutants for PDGF-B, PDGF-Rβ, and PDGF-Rα are lethal due to defects in embryonic development [39]. Knockout of the PDGF-A gene in mice causes a lethal emphysema-like phenotype due to failure of myofibroblast development and subsequent formation of alveolar septum [40,41]. A similar phenotype is seen in genetically partially rescued PDGF-Rα-null mutants [42]. The targeted overexpression of PDGF ligands in the lungs of transgenic mice produces a lethal phenotype associated with hyperplasia of mesenchymal cells [43-46]. Collectively, these transgenic studies indicate that PDGF and its receptors are critical to lung mesenchymal cell survival during pulmonary fibrogenesis.

PDGF and its receptors are potentially important therapeutic targets in pulmonary fibrosis. Because PDGF is a key mitogen and chemoattractant for mesenchymal cells, targeting PDGF or its receptors could be effective in limiting the replication of these cells and reducing collagen deposition and matrix formation. Inhibition of PDGF activity with kinase inhibitors has been demonstrated to significantly reduce lung fibrosis in animal models [47-50]. Imatinib mesylate (Gleevec), an inhibitor of PDGFR tyrosine kinase and c-Abl, has been evaluated in a clinical trial for the treatment of IPF [51]. However, a recent study showed no significant beneficial effect of imatinib on IPF. Agents that downregulate PDGFR expression at the cell surface of mesenchymal cells could also be of potential therapeutic value. For example, PGE_2_, an arachidonic acid metabolite generated by the cyclooxygenase-2 (COX-2) enzyme, is protective in lung fibrosis partly because it downregulates the PDGF-Rα and suppresses fibroblast growth [52]. Unlike TGF-β1, which also downregulates PDGF-Rα, PGE_2 _does not stimulate collagen secretion by fibroblasts. Reduced PGE_2 _results in enhanced epithelial cell apoptosis and yet increases mesenchymal cell resistance to apoptosis [15]. Although COX-2 is a therapeutic target for arthritis, there is considerable evidence that COX-2 serves a protective role in pulmonary fibrosis. For example, COX-2-deficient mice are susceptible to pulmonary fibrosis induced by V_2_O_5 _or bleomycin and produce lesser quantities of PGE_2 _[53,54]. In addition, COX-2 deficiency in mice results in a loss of the antiproliferative response to TGF-β1 [55]. This is further evidence that suggests COX-2 is protective through limiting mesenchymal cell survival.

### The EGF Family: The Duality of Protecting Epithelial and Mesenchymal Cells

The EGF family of ligands mediate numerous cellular activities, including proliferation [56], adhesion [57], migration [58], apoptosis [59] and differentiation [56]. EGF ligands bind to a complex system of cell surface receptors, termed the ErbB system, composed of four membrane-associated proteins, ErbB1 (also known as EGFR), ErbB2 (an orphan receptor), ErbB3 and ErbB4. Like PDGF receptors, each of the ErbB receptors consists of an extracellular ligand-binding domain, a short membrane-spanning region and a cytoplasmic region possessing tyrosine kinase enzymatic activity. EGF ligands include EGF, transforming growth factor-α (TGF-α), heparin-binding EGF-like growth factor (HB-EGF), amphiregulin (AR), neuregulin (NRG), betacellulin, epiregulin and epigen. The EGF ligands bind differentially to the ErbBs and initiate homodimeric or heterodimeric receptor dimerization to cause tyrosine phosphorylation of intracellular receptor residues and downstream cell signaling through mitogen-activated protein (MAP) kinases, phosphatidylinositol 3-kinase (PI3K), and transcription factors including STAT-3 [60,61].

The EGFR ligands are important to epithelial repair following injury, and as illustrated in Figure 3, certain EGFR ligands (TGF-α, HB-EGF) also play important roles in the pathogenesis of pulmonary fibrosis by promoting mesenchymal cell survival and proliferation [8]. Therefore, their role has been described as both protective against acute lung injury or profibrogenic, depending on the context of lung injury or the inciting agent. For example, the administration of recombinant amphiregulin attenuates bleomycin-induced pulmonary fibrosis in mice, suggesting a protective role for this EGFR ligand [62]. TGF-α plays a protective role against nickel-induced lung injury by increasing levels of surfactant proteins [63]. However, the targeted overexpression of TGF-α to distal airway epithelium or conditional expression of TGF-α in mouse lung results in pulmonary fibrosis [64,65]. Alternatively, TGF-α deficiency protects mice from bleomycin-induced fibrosis [66]. Therefore, it is likely that TGF-α exerts its beneficial effects through promoting epithelial repair and increased surfactant production, whereas its profibrogenic activity is most likely linked to its activity as a potent mitogen for mesenchymal cells. In addition, it appears that short-term TGF-α expression stimulates epithelial cell growth and repair during acute lung injury, whereas long term TGF-α expression leads to excessive mesenchymal cell growth and stimulation of matrix deposition and fibrosis. HB-EGF is also a potentially important mitogen for mesenchymal cells. Human airway epithelial cells and human lung fibroblasts both produce HB-EGF in response to vanadium-induced oxidative stress [67,68]. These studies using human cells indicated that paracrine signaling between the airway epithelium and underlying mesenchymal cells as well as autocrine production of HB-EGF by mesenchymal cells could be important to airway fibrogenesis caused by metal injury. Treatment with the EGFR kinase inhibitor AG1478 prior to the instillation of vanadium oxide ameliorates pulmonary fibrosis [47]. Also, AG1478 attenuates upregulation of procollagen expression in tracheal explants from rats exposed to cigarette smoke [69]. Therefore, several lines of evidence indicate that signaling through EGFR is important to both mesenchymal cell proliferation and matrix production during fibrogenesis. However, unlike PDGF family members, which are primarily mesenchymal cell survival factors, EGF ligands are also important survival factors for the lung epithelium and therefore appear to function in both repair following injury as well as disease progression.

### Th1 versus Th2 Inflammation in Mesenchymal Cell Survival and Lung Fibrosis

Although polypeptide growth factors such as PDGF and EGF ligands are important for maintaining mesenchymal cell survival and proliferation, the survival of these cells is also determined in large part by the type of inflammatory microenvironment. Within these microenvironments, mesenchymal cells are bathed in a variety of cytokines, chemokines and lipid mediators that influence cell survival. Some of these factors that modulate mesenchymal cell survival and phenotype are illustrated in Figure 3. Inflammatory reactions are characterized by the infiltration of mononuclear cells including macrophages, lymphocytes, neutrophils and eosinophils. Although inflammation typically precedes fibrosis, evidence from experimental animal models of fibrosis and clinical studies where anti-inflammatory drugs have little effect on lung fibrosis suggest that inflammation may not be required for fibrogenesis [6]. However, the idea that inflammation and fibrosis may be distinct processes is likely an oversimplification, as it is apparent that inflammatory cytokines and chemokines have potent modulatory effects on growth factor activity. For example, during asthma, infiltrating Th2 lymphocytes produce interleukin-13 (IL-13), a key cytokine that mediates multiple phenotypes of airway remodeling, including mucus cell metaplasia, eosinophilia, airway smooth muscle thickening and airway fibrogenesis [8]. IL-13 has also been proposed to play a role in some animal models of interstitial lung fibrosis models, including bleomycin and FITC [70]. Transgenic mice that overexpress IL-13 develop tissue fibrosis through production and activation of TGF-β1 [71]. Studies using a bleomycin-induced pulmonary fibrosis demonstrated that IL-13 signaling through the IL-13α2 receptor is involved in induction of TGF-β1 production and fibrosis [72]. The proliferation of lung myofibroblasts in response to IL-13 is mediated through the autocrine release of PDGF-AA and PDGF-CC [73]. As illustrated in Figure 3, IL-13 generated during a Th2 inflammatory response is important in airway and interstitial fibrosis due in part to its ability to increase PDGF and TGF-β1, which in turn influence mesenchymal cell survival and collagen deposition.

Although IL-13 appears to be central to the pathogenesis of airway fibrosis in asthma and in some animal models of interstitial fibrosis, other models of lung fibrosis are not dependent on Th2 inflammation and IL-13. For example, V_2_O_5_-induced lung fibrosis in mice features Th1 inflammation and elevated levels of interferon-γ (IFN-γ) and IFN-inducible cytokines along with elevated levels of profibrogenic growth factors (PDGF-CC, CTGF, TGF-β1) and collagen with no apparent increases in IL-13 [74]. IFN-γ is a potent Th1 lymphokine that inhibits mesenchymal cell growth and stimulates apoptosis [75]. As illustrated in Figure 3, IFNs play an important role in mediating myofibroblast growth arrest and apoptosis that favors the resolution of a fibrogenic response. Because of the potent growth arrest activity toward normal mesenchymal cells, IFN-γ was investigated and tested in clinical trials as a potential antifibrotic therapeutic agent. Although initial preliminary studies indicated antifibrotic potential [76], a blinded follow-up study showed no consistent beneficial effects of IFN-γ on the survival of IPF patients [77]. This could be due to the refractive nature of a well-established collagen matrix that comprises end-stage fibrotic lesions or other properties of IFN-γ that influence the progression of fibrosis. For example, although IFN-γ is antimitogenic toward lung fibroblasts, it also enhances particle-induced PDGF production by alveolar macrophages [78] and enhances the proliferative activity of PDGF and EGF for lung fibroblasts isolated from mice deficient in the STAT-1 transcription factor [79].

In addition to IFN-γ, the classic proinflammatory cytokines IL-1β and TNF-α are increased in V_2_O_5_-induced lung fibrosis in mice and rats [53]. A variety of fibrogenic agents, including particles and fibers, increase the secretion of IL-1β by alveolar macrophages [80]. IL-1β has been shown to increase the production of PDGF by mesenchymal cells [81] and is also a potent inducer of the PDGFRα on rat lung myofibroblasts [82]. IL-1β overexpression in mice causes pulmonary fibrosis [83], and more recent work shows that IL-1β enhances bleomycin-induced fibrosis by upregulating IL-17A [84]. Although IL-13 was also upregulated in this study using the bleomycin model, its expression was at a relatively late stage and occurred after collagen deposition. Nevertheless, it is likely that IL-13 contributes to chronic interstitial pulmonary fibrosis by promoting mesenchymal cell survival.

Overlapping Th1 and Th2 inflammatory responses can occur when individuals with allergic asthma are exposed to agents that normally elicit only a Th1 inflammatory response. In this case, the mixture of IL-13 and IFN-γ are largely antagonistic, where IL-13 promotes mesenchymal cell survival and IFN-γ inhibits mesenchymal cell growth and stimulates apoptosis. However, IL-13 and IL-β can act coordinately on rat lung myofibroblasts to enhance their proliferation. For example, the effect of IL-13-induced PDGF-AA production by rat lung myofibroblasts is further amplified by IL-1β, which upregulates the PDGF-Rα [85]. Carbon nanotubes or V_2_O_5 _elicit a Th1 inflammatory response in the lungs of mice or rats, characterized by increased levels of IFNs and IFN-inducible chemokines, as well as PDGF [23,74]. In mice that develop an allergic airway Th2 inflammatory response induced by ovalbumin challenge, carbon nanotube exposure synergistically increases airway fibrosis [22]. In this case, the combined effects of Th1 and Th2 inflammation resulted in an enhanced fibrogenic response.

### STAT Transcription Factors as Mediators of Mesenchymal Survival

**Figure 4:**
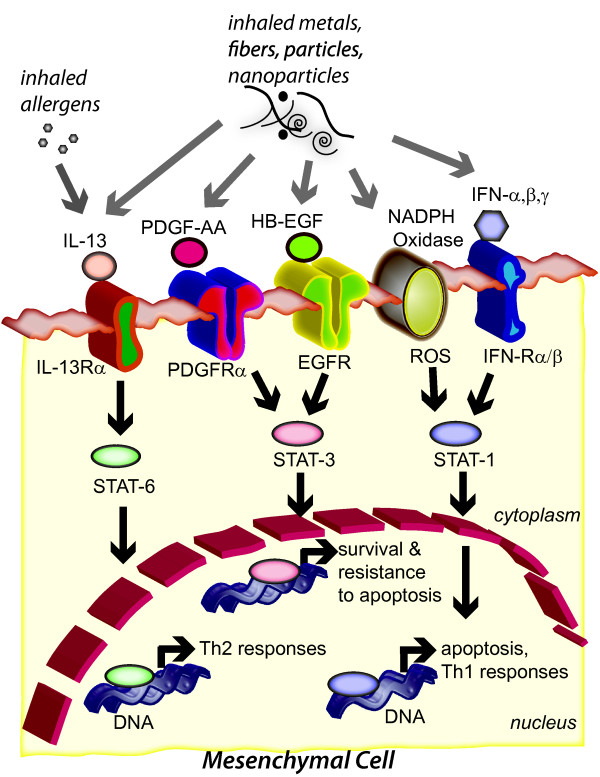
**STAT transcription factor signaling outcomes in mesenchymal cells that contribute to pulmonary fibrogenesis**. Multiple signals, either endogenous factors (cytokines and growth factors) or environmental factors (metals, particles, nanoparticles), activate STAT signaling that leads to outcomes involved in fibrogenesis and tissue repair. IL-13 is increased by allergens or certain metals and particles to activate STAT-6, which in turn results in Th2 inflammatory responses that include the production of profibrogenic growth factors, PDGF-AA and TGF-β1. These growth factors are also increased by metals, particles and nanoparticles. PDGF-AA and HB-EGF stimulate STAT-3 to turn on a mesenchymal cell "survival program." Metals and particles also increase the production of reactive oxygen species (ROS) through NADPH oxidase activity or increase the production of interferons (IFNs). ROS or IFNs stimulate STAT-1 to promote growth arrest and apoptosis of mesenchymal cells. Therefore, the proapoptotic action of STAT-1 opposes the prosurvival and antiapoptotic actions of STAT-3 and STAT-6 for mesenchymal cells.

Many of the cytokines and growth factors mentioned above that regulate mesenchymal cell survival or mesenchymal cell growth arrest and apoptosis act via a family of transcription factors termed the signal transducers and activators of transcription (STATs) [86,87]. Some of the possible STAT-dependent signaling outcomes that occur in mesenchymal cells that influence the progression or resolution of lung fibrosis are illustrated in Figure 4. STATs were originally identified because of their ability to transduce signals from a cellular receptor into the nucleus and thereby modulate the transcription of specific genes. Upon ligand binding, receptor kinases activate latent cytoplasmic STATs through tyrosine phosphorylation [88]. The STAT proteins then homo-or heterodimerize and translocate to the nucleus, where they bind to DNA and modulate gene expression. STAT family members bind with varying affinities to a canonical palindromic sequence (TTCN_2-4_GAA) in the promoters of their target genes [89]. STATs play prominent roles in both pro-and anti-inflammatory processes, including cell proliferation, apoptosis and differentiation. In the context of this review, STATs are pivotal in mediating both mesenchymal cell survival and mesenchymal cell death.

Interferons (IFNs) are important in resolving fibrogenesis and activate STAT-1 signaling pathways for mesenchymal cell growth arrest and apoptosis. Transcriptionally active STAT-1 is required for the antiproliferative and proapoptotic effects of IFNs on mesenchymal cells [87]. Therefore, STAT-1 is central to mediating the effects of IFNs in the lung by regulating mesenchymal cell growth arrest and apoptosis, which favors the resolution of a fibroproliferative response. STAT-1^-/- ^mice show no overt developmental abnormalities but display a complete lack of responsiveness to either IFN-γ or IFN-α and are susceptible to infection by microbial pathogens [90,91]. However, STAT-1^-/- ^mice develop more severe pulmonary fibrosis after lung injury with bleomycin [79]. This study indicated that STAT-1^-/- ^mice are more susceptible than wild-type mice to bleomycin-induced lung fibrosis owing to (1) enhanced fibroblast proliferation in response to growth factors (EGF and PDGF), (2) stimulation of fibroblast growth by a STAT-1-independent IFN-γ signaling pathway, and (3) increased activation of STAT-3. PDGF-BB or EGF have significantly greater proliferative effects on fibroblasts isolated from the lungs of STAT-1^-/- ^mice compared to wild-type (STAT-1^+/+^) mice [79]. Moreover, STAT-3 activation in response to PDGF or EGF, a prosurvival signaling event for mesenchymal cells, is significantly greater in STAT-1^-/- ^mouse lung fibroblasts compared to STAT-1^+/+ ^fibroblasts. These findings indicate that STAT-1^-/- ^mice are more susceptible to bleomycin-induced lung fibrosis than STAT-1^+/+ ^mice owing to enhanced fibroblast proliferation in response to growth factors (PDGF and EGF) and increased activation of STAT-3. In addition, IFN-γ has a proliferative effect on fibroblasts isolated from the lungs of STAT-1^-/- ^mice, whereas IFN-γ is growth inhibitory to fibroblasts isolated from the lungs of wild-type STAT-1^+/+ ^mice [79]. These findings indicate that IFNs exert dual antimitogenic effects via STAT-1 and promitogenic effects via STAT-1-independent signaling pathways. This dual action may explain why IFN-γ has not proven to be an effective therapy in patients with IPF [77]. In addition to studies showing that deletion of STAT-1 potentiates bleomycin-induced lung fibrosis in mice, other work demonstrated that aerosolized STAT-1 antisense oligodeoxynucleotides decreased the concentrations of TGF-β, PDGF and TNF-α in bronchioalveolar lavage fluid (BALF) in bleomycin-induced rat pulmonary injury and ameliorated bleomycin-induced pulmonary fibrosis [92]. Finally, more translational work with human lung fibroblasts shows that IFN-γ inhibits TGF-β1-induced signaling and collagen production via STAT-1 [93]. All of these studies clearly indicate that STAT-1 plays a protective role in limiting mesenchymal cell survival and resolving lung fibrosis. Furthermore, the development of novel agonists that activate STAT-1 may prove beneficial for managing or treating pulmonary fibrosis.

While STAT-1 is principally activated by IFNs through their cognate cell-surface receptors on mesenchymal cells, reactive oxygen species (ROS) are also capable of activating STAT-1 [94]. A variety of environmental factors (e.g., metals, air pollution particulate matter, nanoparticles, cigarette smoke, ozone) generate ROS that activate intracellular signaling cascades. For example, STAT-1 activated by the transition metal V_2_O_5 _is blocked by anti-oxidants N-acetyl-L-cysteine or catalase [95]. More recent findings showed that STAT-1 activation in human lung fibroblasts by V_2_O_5 _required NADPH oxidase-generated ROS and autocrine production of IFN-β [96]. This resulted in antifibrogenic signals, including growth inhibition but also the increased expression of the IFN-inducible chemokine CXCL10. CXCL10 is a pleiotropic molecule that elicits potent biological effects, including chemotaxis of activated T and NK cells, modulation of adhesion molecule expression, and inhibition of angiogenesis [97,98]. CXCL10 reduces bleomycin-induced pulmonary fibrosis in mice via inhibition of angiogenesis [99]. Deletion of CXCR3, the receptor for CXCL10, increases bleomycin-induced fibroproliferation and mortality in mice [100]. Therefore, our findings support the hypothesis that STAT-1, IFNs and CXCL10 are protective factors in the lung that limit the severity of a fibrogenic response and promote the resolution of fibrosis. These events act in opposition to and occur after the profibrogenic actions of V_2_O_5 _in mice and rats that results from increased expression and activation of profibrogenic growth factors such as PDGF, TGF-β1, and CTGF.

Whereas STAT-1 plays a key role in promoting apoptosis in a variety of cell types and has antiproliferative effects, STAT-3 acts in opposition to STAT-1 and has an antiapoptotic effect and promotes mesenchymal cell proliferation [101,102]. In contrast to deletion of STAT-1 or STAT-6, STAT-3 deletion in mice is lethal and therefore little is known about the role of STAT-3 in lung fibrosis. STAT-3 is generally thought to promote the survival of lung mesenchymal cells in response to growth factor stimulation [101]. Fibroblasts isolated from normal human lung do not proliferate in response to IL-6 due to prolonged STAT-3 signaling, whereas fibroblasts from IPF patients proliferate in response to IL-6 [103]. This mechanism involved a shift in signaling dependency from STAT-3 in normal human fibroblasts to ERK in IPF fibroblasts. While STAT-3 deletion in mice is lethal, the selective deletion of STAT-3 gene in respiratory epithelial cells by conditional expression of Cre-recombinase under control of the surfactant protein C gene promoter did not alter prenatal lung morphogenesis or postnatal lung function [104]. However, exposure of adult STAT-3-deleted mice to hyperoxia caused a more rapidly progressive lung injury associated with alveolar capillary leak and acute respiratory distress, suggesting that STAT-3 plays a critical role in maintenance of surfactant homeostasis and lung function during oxygen injury in adult lung tissue.

STAT-6 is activated by Th2 cytokines such as IL-13 and IL-4, but not by polypeptide growth factors such as PDGF and EGF that mediate mesenchymal cell survival. However, as mentioned above, these growth factor families are induced by IL-13 and this signaling is accomplished through STAT-6 [8]. STAT-6 mediates many of the biological effects of IL-13 during asthma pathogenesis and fibrosis. All of these characteristics of airway remodeling in asthma (eosinophilia, mucous cell metaplasia, airway fibrogenesis) are absent in a model of allergic asthma in STAT-6-deficient mice [105]. A primary role for IL-13 in asthma and Th2-mediated fibrogenic reactions is the production of TGF-β1 via a STAT-6-dependent mechanism [71,72]. STAT-6 also mediates IL-13-induced production of PDGF-AA in rodent and human lung fibroblasts [73]. Therefore, STAT-6 plays a central role in orchestrating the expression of profibrogenic growth factors during allergic lung diseases and fibrosis. While STAT-6 is the primary signaling intermediate for the biological effects of IL-13, STAT-1 is also activated by IL-13 in a variety of lung cell types [106]. However, STAT-1 antagonizes IL-13-induced signaling in lung cell types [107,108]. Therefore, a common theme is that STAT-1, activated by IFNs, antagonizes STAT-6 and STAT-3 to exert opposing biological effects mediated by IL-13 or growth factors, respectively.

## Conclusions

Lung fibrosis encompasses a wide spectrum of diseases and disorders that are initiated and perpetuated by a complex interplay of genes and environment. Despite the diversity of causes for fibrosis and the multiple mechanisms that initiate the disease process, a common denominator that is pivotal to disease progression is survival of mesenchymal cells. Nevertheless, current treatment strategies have not been effective in preventing or managing pulmonary fibrosis. Apoptosis of fibroblasts is required for successful wound healing and termination of collagen deposition [70], and resistance to apoptosis has been observed in fibroblasts from IPF patients [103]. Therefore, promoting mesenchymal cell apoptotic pathways at the appropriate time after lung tissue repair may help slow the progression of fibrosis. Targeted therapy aimed at growth factors (e.g., PDGF or TGF-β1) and their receptors to limit mesenchymal cell survival and collagen deposition seems a logical path for the treatment of fibrosis, given the important roles that these growth factors play in mesenchymal cell survival and collagen production. However, while growth factor tyrosine kinase inhibitors showed promising results in attenuating lung fibrosis in experimental animal models, recent studies with kinase inhibitors (e.g., imatinib mesylate or Gleevec) have shown no effect on the survival or lung function of patients with IPF [109]. Likewise, clinical trials with IFN-γ, which also showed promising results in animal models of pulmonary fibrosis, have failed to show any significant beneficial effect in IPF patients [77]. As discussed in more detail above, IFN-γ is clearly growth inhibitory to mesenchymal cells through STAT-1 signaling, but there is also evidence that indicates IFN-γ can promote mesenchymal cell survival through STAT-1-independent signaling [79]. It has been suggested that animal models of pulmonary fibrosis (e.g., bleomycin) do not adequately model IPF. However, fibrotic reactions in IPF patients undergoing treatment with IFN-γ or imatinib are relatively end stage after much tissue scarring has occurred, and interfering with mesenchymal cell survival at this point may simply come at a stage that is too late to be effective. Imatinib therapy might be effective in the early stages of fibrogenesis as in patients undergoing lung transplant who suffer a high incidence of bronchiolitis obliterans [110]. Some anticancer therapies, such as those targeting erbB2 (an EGF receptor family member) with monoclonal antibodies, might be considered for lung fibrosis therapy to reduce mesenchymal cell survival and resolve a fibrotic reaction. Finally, antifibrotic drugs tested in the future could be more efficiently administered to target tissues via nanoparticle-mediated drug delivery, although some caution should be used as some nanoparticles exacerbate airway fibrotic reactions in mouse models of allergic asthma [111]. Mesenchymal survival remains an important issue, and further research toward controlling the survival of these cells should eventually result in the development of effective treatments for lung fibrotic diseases.

## Competing interests

The author declares that they have no competing interests.

## Authors' contributions

JCB drafted the manuscript in its entirety, designed all original figures, and approved the final edited manuscript.
